# Decision making and support available to individuals considering and undertaking electroconvulsive therapy (ECT): a qualitative, consumer-led study

**DOI:** 10.1186/s12888-018-1813-9

**Published:** 2018-07-24

**Authors:** Karen Wells, Justin Newton Scanlan, Lisa Gomez, Scott Rutter, Nicola Hancock, Anthony Tuite, Joanna Ho, Sarah Jacek, Andrew Jones, Hassan Mehdi, Megan Still, Graeme Halliday

**Affiliations:** 1 New Horizons Inc, Ashfield, Australia; 2 The University of Sydney, Faculty of Health Sciences, PO Box 170, Lidcombe, NSW 1825 Australia; 3 Sydney Local Health District, Mental Health Services, Concord, Australia

**Keywords:** Electroconvulsive therapy, Consumer-led research, Service user research, Qualitative methods, Patient experience

## Abstract

**Background:**

Electroconvulsive therapy (ECT) is one of the most controversial treatments in psychiatry. This controversy and diverse and often strongly held opinions can make decision making processes around ECT more complex.

**Method:**

This consumer-led project explored the experiences of individuals who had received ECT in terms of the information they received, their experience of ECT and suggestions for ways that decision making processes and experiences of ECT can be improved. Interviews were conducted by consumer researchers who had also received ECT and transcripts were analysed using constant comparative techniques.

**Results:**

Seventeen individuals participated. Four overarching categories were identified from participant interviews*: Information matters*; *Preparation and decisions before ECT*; *Experience of ECT*; and *Suggestions for improvement.* Most participants suggested that more information was required and that this information should be made available more regularly to support decision making. Additional suggestions included greater involvement of family and friends (including having a family member or friend present during the ECT procedure), opportunities to gain information from individuals who had received ECT and more support for managing memory and cognitive side effects.

**Conclusion:**

This study provides valuable consumer-provided insights and recommendations for psychiatrists and mental health clinicians working within ECT clinics and with consumers considering or preparing for ECT.

**Electronic supplementary material:**

The online version of this article (10.1186/s12888-018-1813-9) contains supplementary material, which is available to authorized users.

## Background

Electroconvulsive therapy (ECT) is arguably the most controversial treatment in modern psychiatry [1, 2]. Advocates argue that it is a safe and lifesaving intervention. Opponents suggest it is ineffective [3] and some go so far as to suggest that ECT is “a crime against humanity”, calling for it to be banned (MINDFREEDOM, cited in [4], p. 17). Community attitudes are also influence by images of ECT presented in popular culture which can include scenes of individuals being strapped to tables and fitting violently.

Evidence for the effectiveness of ECT is contested. Some suggest that ECT is effective in up to 85% of individuals with severe depression [5]. It has also been suggested that ECT can support positive outcomes more quickly than medications or other interventions [6, 7] and reduce rates of suicide [6]. However, others conclude that there is no evidence-based research that demonstrates ECT is better than a placebo (where anaesthetic is given to the patient, but no electric current is applied) beyond the treatment period [8]. Further criticisms are that there have been no placebo-controlled studies of ECT since 1985 [9] and the claims that ECT prevents suicide cannot be substantiated [9].

The long-term side-effects of ECT are also contested, especially in relation to memory. Some state that ECT has mainly temporary effect on memory and some even suggest that memory improves as a result of depressive symptoms improving [10, 11]. However, others assert that memory loss is common [12, 13] and some contend this is the result of irreversible brain damage [3, 8].

Due to the diversity of information available, when ECT is raised with individuals as a potential treatment, finding reliable, balanced information can be challenging. This was described as: “My attempt at gathering accurate information went far beyond a typical patient’s, yet I gained little more than the fear and confusion generated by grossly conflicting and limited data” ([14], p., 140).

While a large volume of ECT-related research exists, including many studies of participants’ experiences of receiving ECT [15], fewer studies have specifically explored the information and support needs of people considering ECT. This is especially true in terms of research led by consumers: individuals with lived experience of mental illness, and in this case, of receiving ECT.

Some previous consumer-led research has explored the area of ECT (e.g., [12, 16, 17]). These studies have generally reported more negative results than those led by health professionals [17]. Potential reasons for this discrepancy include: (i) consumers may feel freer to discuss their experiences with other consumers; (ii) participant selection processes may differ; (iii) qualitative techniques have tended to be used and may have more effectively uncovered these negative experiences; and (iv) the majority of this research has been completed at later stages of people’s experiences when initial effects have worn off and persisting issues have become more apparent [18].

The diverse and often strongly held [19] opinions about ECT and the controversial nature of the topic [1, 2] may inhibit open discussion. Without full and open conversations, individuals are less likely to be able to make the best-informed decisions about ECT. Therefore the “ECT – Let’s talk about it!!” project was established. The primary aim of this collaborative, consumer-led project was to explore individuals’ experiences of ECT; what helped or hindered their decision making process; and what could improve the decision-making process and the overall experience of ECT.

## Method

This study was approved by the participating hospital’s Human Research Ethics Committee. The research team consisted of the project lead, a project officer and two consumer researchers. All of these individuals had lived experience of ECT. Each member’s experience of ECT differed: from negative experiences (that ECT was ineffective and traumatic), through to very positive experiences (that ECT had saved their life). A qualitative approach was selected to gain in-depth understanding of this little-known topic. Researchers engaged in individual and group reflective processes throughout the study to ensure personal perspectives did not influence the project.

In line with calls for more “genuinely collaborative” research between consumers and health professionals about ECT [20], the consumer research team engaged a steering committee including members from hospital-based mental health services and a local non-government organisation as well as two experienced researchers (without lived experience of mental illness) from a local university. The steering committee assisted with the design and implementation of the project (although the final decision making authority remained with the consumer research team) and the university-based researchers assisted with research design, training and data analysis. This collaborative form of consumer-led research has been suggested to enhance the overall research process [16, 21].

### Sampling and recruitment

Inclusion criteria were the ability to communicate in English and previous experience of ECT. Participants were recruited through advertisements placed in ECT clinics, community mental health services, newsletters, conferences, advocacy groups and via mental health clinicians. All participants provided written, informed consent and received a gift voucher as a token of appreciation. Recruitment ceased when data saturation was achieved (i.e., no further concepts were being gained from further interviews and categories were rich and well described).

### Data collection

Interviews were conducted by consumer researchers (authors LG and AT) and followed a semi-structured format, aided by use of an interview guide (see Additional file 1). The guide was flexible and modified to include additional topics raised in earlier interviews. Interviews were completed at the host hospital, community mental health services or via telephone. Interviews took between 30 to 60 min and were audio recorded and transcribed verbatim.

### Data analysis

Data analysis occurred concurrently with data collection in an iterative process. Data were analysed using constant comparative analysis [22] to systematically compare data within and between interview transcripts. Constant comparative analysis is an inductive approach, with codes developed from the data itself, rather than from the use of a pre-determined coding frame [22, 23].

Initially, the four members of the consumer research team (KW, LG, SR and AT) and a university researcher without lived experience of mental illness (JNS) read and coded the first three interview transcripts and developed a preliminary set of codes. They then met together to discuss and find consensus in the early coding decisions. Following on from this initial coding, focused coding involved the drawing together of conceptually equivalent codes into broader themes. Throughout the data analysis process, regular reflexive discussions between the coding team (KW, LG, JNS) with additional guidance from another university researcher (NH) ensured emerging themes were representative of the data [22]. The data analysis process did not involve members of the steering committee to ensure that the consumer led nature of the study was not compromised.

## Findings

Seventeen individuals participated. Demographic characteristics are summarised in Table 1.

**Table 1:** Demographic characteristics of participants

Characteristic	n (%)
Gender	
Male	10 (59%)
Female	7 (41%)
Age	
Mean (Range)	54 years (21 to 85 years)
Diagnosis	
Depression	9 (53%)
Bipolar disorder	3 (18%)
Schizophrenia	2 (12%)
Unsure	3 (18%)
Course	
Within past 5 years	10 (59%)
More than 5 years ago	5 (29%)
Unsure	2 (12%)
Type of hospital (first ECT)	
Public	10 (59%)
Private	6 (35%)
Unsure	1 (6%)
Number of courses	
Only one course	7 (41%)
Multiple courses	8 (47%)
Unsure	2 (12%)
Time since last course	
Current / within 3 months	6 (35%)
Within past 2 years	7 (41%)
Unsure	4 (24%)

Four themes were identified. These were: *Information matters*; *Preparation and decisions before ECT*; *Experience of ECT*; and *Suggestions for improvement*.

### Information matters

Participants highlighted the importance of understanding what would be involved prior to their ECT treatment. The information they received came from various sources and either facilitated or hindered their understanding.

#### Accessible and open treating team

All participants, except one who raised the option of ECT with her treating team following her own research, reported that their doctor (and occasionally other members of the treating team) was the primary source of information about ECT. Satisfaction with the amount of information provided varied. Satisfied participants were provided with information in various formats: “psychiatrist explaining it to you, and you had the video and pamphlets - that’s pretty comprehensive” (P17). These participants also talked about the accessibility and openness of their treating team to providing information: “We could ask any questions... That was very valid[ating] actually, to have a conversation with someone that could answer your questions…” (P7).

However, this open and comprehensive information sharing from the treating team was not experienced by all participants. P15 said: “I just remember feeling terrified… I just think there could [have] been more information; that would have calmed me”.

#### Seeking variable information from other sources

Six participants talked about seeking information about ECT from other sources, via the internet and reading books. Sometimes this search resulted in finding information that they considered helpful and made them feel more comfortable about ECT: “I did read an article where it said, ‘This is a benign procedure really’, and comparing it to the destruction of depression and the risks with that. It made it seem more positive, so that was helpful” (P15). One participant raised ECT as an option with her treating team after her own research: “I think I Googled it honestly. I probably just read about it on Wikipedia or something. I saw the success rate was pretty high, so I might as well just bring it up and see if it’s an option” (P12).

However, some of the information discovered in these searches raised concerns for participants. P8 commented: “the impression I had been given from reading… was that it was something horrifying” (P8). One participant intentionally stopped seeking information because of the fear it might induce: “I knew that a lot of the information around was very negative and scary. I didn’t want to find out too much about it and put myself off” (P12).

Other sources of information were knowledgeable family members and others who had received ECT.

### Preparation and decisions before ECT

Beyond information, participants described a range of other factors that influenced their preparation for, and decision to have ECT when they had, and chose to engage with, that choice. Some factors enhanced this preparation and decision making process while others detracted.

#### Factors that made it more likely for individuals to undertake ECT

Numerous factors resulted in participants being more likely to choose, agree or accept having ECT, or enhanced their preparation experience. These included: the doctor made the decision; a desperate last resort; witnessing others or myself benefitting; and family support.

##### The doctor made the decision

Many participants (*n* = 10) described that it was their doctor who made the final decision for ECT. For some, this involved putting their faith in their doctor: “I think it was just the trust in him really. It was really my only hope…” (P15).. Others, often overwhelmed by the decision making process, allowed the treating team to make the decision: “I went along with it” (P2) and “I had no reason to dispute what the [treating team’s] ideas were” (P9). For other participants, they reported limited input into the final decision: “I think you have to be positive even if they are making up your minds for you, you have got to think positive and be a good patient, which I think I am” (P8).

##### A desperate last resort

For nine participants, the choice for, or acceptance of ECT, came from a place of desperation or the sense that there were no other options. P12 said: “it felt like my last chance to do something because I thought, ‘If that doesn’t work, I don’t know what I’m going to do…this is the last resort and this is my only option. Whatever happens, I have to accept as the risk I take trying to get better.’” P7 said: “… if a doctor… told me, ‘put your head in the toilet and I’ll flush it and your pain will go’ I probably would have done it… That’s how bad I was feeling”.

##### Witnessing others or myself benefitting

Three participants were encouraged by witnessing other people’s mental health improvement following ECT. “I’d seen how it helped the young girl in [hospital] a couple of years before, so I had that in my head, like, ‘Okay, I’ve seen it work for someone’” (P17). Four participants also described their own previous experiences of ECT. When they had previously experienced it as effective they were more likely to accept future ECT treatment. For example, P15 was accepting of further ECT “as it saved my life. I wouldn’t have been able to continue [without it]”.

##### Family support

Eight participants described being more accepting of ECT when their family were encouraging or positive about it. “They automatically thought it was a good idea to go on ECT. That’s how I came to be on it” (P5).

#### Factors that made it less likely for individuals to undertake ECT

Other factors resulted in participants being less likely to choose, agree or accept having ECT, and/or negatively impacted on their preparation experience. These included fear, negative family experiences and concerns about side effects.

##### Fear

Repeatedly, participants discussed the fear they experienced prior to ECT. Six participants described quite strong fears prior to ECT, particularly a fear of dying during the procedure. P3 said, “You don’t know what’s going on… you might die under the treatment. I… was worried” and P10 said “What if I have a heart attack during that and died?”

##### Negative family experiences

Previous negative experiences of family members were also influential. Five participants had a family member who had experienced ECT, with four participants describing these experiences as quite negative. “My father had had it… back in the ‘50s... I was horrified about the whole idea of it… I suppose it was the fear from my father’s time... Now here I was facing the same thing” (P15). P4 said: “one thing that worried me were my father’s memories of his mother having ECT and they weren’t that good”.

##### Concerns about side effects

Concerns about side effects were repeatedly discussed by participants. Three participants talked specifically about significant concerns about memory loss. “I was very worried about memory loss actually. That was the main thing that I read about. That’s the thing that I was worried about” (P12). These concerns were heightened by seeing others going through ECT: “…her memory was really bad. For a day afterwards, she was very lethargic and very worn out… seeing her side effects made me think about it a lot more” (P17).

### Experience of ECT

Participants described both positive or helpful and negative or unhelpful aspects of their experience.

#### Positive aspects

Numerous aspects enhanced participant experiences of ECT. These included: sharing the journey with others; caring and compassionate interactions with staff; being less scary than expected; and rapid mental health improvements.

##### Sharing the journey with others

Three participants spoke of the value of sharing experiences with other people who were also having ECT. For example, P17 said: “we started ECT around the same time… you got to talk to someone who actually did have that treatment the morning before. We were definitely on that little journey together, which was really helpful”.

##### Caring and compassionate interactions with staff

Staff were another source of comfort. Four participants commented on caring staff members’ ability to easy their anxiety and make the process more comfortable. Referring to staff involved in ECT sessions, P12 said “I was really surprised… a lot of the people there were really nice”. P2 said: “they treat you more like a person rather than an object. I think there is some level of compassion and acceptance and respect”.

##### Being less scary than expected

While many participants reported feeling fearful about ECT, six participants commented that it was easier and less scary than they expected.P15 commented that “fears dropped away” after ECT had commenced and P17 said: “I was very anxious and… scared. It was just the unknown. As it went on… I got less scared and more hopeful”.

##### Rapid mental health improvements

A final positive aspect of the process for many was that improvements came quickly, bringing relief from extreme suffering. Seven participants specifically commented on the relief experienced after receiving ECT, especially after a long period of being unwell. P15 said: “I was getting such a good result straightaway… and getting better finally” (P15).

#### Negative aspects

While participants described positive aspects of the ECT process, there were also numerous negative aspects. These included side effects; aspects of the procedure itself; stigma and shame; and waiting in line.

##### Side effects

Participants reported numerous side effects, particularly side effects related to memory and cognition. Sixteen of the 17 participants discussed memory or other cognitive difficulties. Six of these participants described significant memory difficulties. For example, P7 said: “A lot of things that happened before I went in seemed to be almost entirely wiped from my memory” and P15 reported “that’s been hard to adjust to: that maybe I lost some of my cognitive capacity”. In terms of cognition, P17 said “It’s affected my concentration… things don’t come to me as quickly. Things don’t flow as much”.

##### Aspects of the procedure itself

Five participants reported feeling worried or distressed about specific aspects of the ECT process. These related to fears of “losing… control” (P16), or concerns about general anaesthetic “…you might die under the treatment” (P3) and not liking needles: “Getting the injection and them putting it in the vein… I didn’t like that” (P10).

##### Stigma and shame

Six participants described a sense of shame about receiving ECT. Grappling with how to discuss ECT with friends, P12 asked: “How do I explain to them that I stopped talking to them because I was depressed and that I’ve had ECT and now I feel fine again? Can you tell people you’ve had ECT? What happens then?” P15 had chosen to not talk to her children about her ECT: “my children, obviously, know I had a mental illness and was very sick, but I haven’t mentioned ECT. I think, unfortunately, I would really find that hard to tell them because it does seem so extreme and frightening for most people”.

##### Waiting in line

Four participants described that waiting in line for ECT was distressing. It increased their anxiety: “of course it’s hard to wait in the line of people… It makes you think about the treatment more, and waiting is… stressful” (P1). Additionally, while participants talked about the comfort peers could provide, witnessing distress of other patients could heighten their own anxiety. “Some people can just be panicky about the waiting… this young lady… who had never done ECT before… she basically freaked out and fled” (P4).

### Suggestions for improvement

Participants offered many suggestions for how to improve the process of supporting individuals in making decisions related to ECT and the treatment experience itself. Suggestions included: more information; bringing it up earlier; information and support from others who have had ECT; better planning for management of side effects; and greater family involvement.

#### More information

Having access to “more information” in a range of formats was the most prominent suggestion made, with nine participants offering suggestions for additional information that should be provided. Two participants suggested that watching the procedure on video would help to clarify any misperceptions: “…see a real one on video. ... and just [be] comforted that it’s really not so frightening” (P15). Participants also suggested more frequent information presented in easy to understand language. “I think I’ve got slightly above average understanding of it and I still don’t understand half of what they are telling me…” (P4). Brief, clear information was also important: “concise, not lengthy, because the person who may be deciding whether to have ECT or not is probably anxious and depressed, so you don’t want to bombard them with too much information” (P2).

#### Bringing it up earlier

Some participants said that leaving discussions and information about ECT until the last minute or until it was considered the last resort compounded an already troublesome decision making process. Three participants specifically identified that it should be raised earlier in the treatment process. P12 suggested that raising ECT earlier as “another treatment option” would help de-stigmatise it and might mean that extended suffering is avoided. P12 said: “If only somebody had said something or if I had known a bit more about it or been brave enough to mention it myself, how different would my experience over the past decade have been?” P17 had similar thoughts: “I’ve seen people and myself that have been unwell for so long, and I think to myself, ‘What if the psychiatrist had suggested ECT to them, would they have gotten more well more quickly?’”

#### Information and support from others who have had ECT

Four participants suggested that hearing information from individuals who had previously received ECT would be helpful. P15 said: “I do sometimes wonder ‘Would it be helpful for someone who has recovered to go into the hospital and talk to people?’ Yeah, I think so”. Participants also suggested that a peer-to-peer support group would be helpful: “Just a group where people can go along and talk about their experience with ECT…” (P17).

#### Better planning for management of side effects

While many participants identified memory and cognitive difficulties as an issue, two participants specifically raised the importance of providing more information and advice about managing side effects that may emerge, especially memory problems. “I wish now that I had paid more attention to the information that I read about memory because it was very different from the information that I was given by my doctor… there doesn’t seem to have been any plan put in place for what would happen if my memory was bad” (P12).

#### Greater family involvement

Finally, two participants specifically raised the importance of family members being more involved throughout the process. One participant even suggested having a family member present in the room when ECT was being performed: “I did watch a video where the mother seemed to be standing beside the bed of the patient, so I don’t know whether that’s an option for people. It doesn’t have to be so sterile and on your own... I think that might be helpful to have someone beside you” (P17).

## Discussion

This study explored participants’ decision making processes and experiences of ECT. Four themes emerged from data analysis: *Information Matters*; *Factors that influenced preparation and decision making*; *The actual experience of ECT*; and *Suggestions to improve the overall experience of ECT*.

While participants in this study generally reported that ECT was helpful, many of them identified that more information was required. This is consistent with a range of previous literature [15, 17]. Limited access to high quality, balanced information may impede the process of fully informed and active decision making. This is also reflected in the number of participants who reported that they were relatively passive in the decision making process. This is highlighted by the category of *The doctor made the decision.* While some participants described a deep sense of trust in their doctors, others felt unable to make a choice or were not involved in the choice. Each of these situations represents an opportunity for consumers to be more fully engaged in the decision making process. Additionally, Ejaredar and Hagen [24] suggested that the urgency with which decisions around ECT often have to be made disempowers consumers and may lead to more instances of potentially coercive decision making. This is also prominent in the voices of participants in this study, with nine reporting that decision making was influenced by a sense of desperation or that nothing else had worked, and is consistent with findings from other research [25–27]. This sense of desperation and urgency may undermine active decision making.

A novel finding from this study was participants’ suggestions that ECT should be raised as a potential treatment option earlier in the treatment process. Participants suggested that this may help to reduce the stigma associated with ECT when it is presented as one of numerous treatment options available. These participants suggested that the lack of discussion of ECT until later in the treatment process resulted in an unnecessarily long period of suffering. Raising ECT as a potential treatment earlier in the process is also likely to enable individuals to do their own research and seek out additional information in a more planned, proactive way and may reduce the impact that a sense of desperation has on the decision making process.

Another important finding from this research was the number of participants who identified significant memory problems. Six participants reported specific challenges with memory and a number of individuals found it difficult to provide information regarding specific information about their treatment (as highlighted by the number of “unsure” responses in Table 1), which may also highlight memory difficulties. These findings are consistent with findings from consumer led studies and testimonials that suggest memory problems are more common than is sometimes suggested [15, 17]. Some participants in the current study felt that potential side effects related to memory and cognition were minimised and that more should have been done to explain these potential side effects and provide access to strategies and resources to manage these challenges if they emerged. This concurs with the call from other authors for greater discussion of the potential for memory problems associated with ECT and the need to provide access to cognitive rehabilitation interventions [1, 14]. It should be noted that for some participants in this study, even very substantial memory loss was considered “worth it” given the relief afforded by ECT. This is consistent with findings from numerous other studies where individuals report their willingness to make “trade-offs” between the troublesome effects of ECT and their perceptions of the benefits gained from it [6, 17].

Participants in this study also commented on the stress created by “waiting in line.” This is similar to findings from Ejaredar and Hagen [24] where participants spoke of “It was like we were cattle,” although participants in the current study spoke more of how it was anxiety-provoking rather than dehumanising.

Additionally, participants highlighted the importance of increasing support from both families and peers. In line with previous studies [28] some participants identified their desire for families and close friends to be more involved in the process. Many participants also commented on the value of being able to discuss ECT with other consumers.

### Some potential ways to improve the consumer experience of ECT

Using the information provided by participants in this study, as well as information from the literature, a range of suggested “ways forward” have been developed.

#### A potential role for peer workers

Considering the diversity of additional needs described by participants, it may be useful for services to consider the potential role for engaging peer workers with a specialisation around ECT. A peer worker with lived experience of ECT could follow up with individuals who are considering ECT and their families to explore if further information is required. Coming from an individual with lived experience, this information may be considered to be “more balanced” [29, 30]. This follow up information would be able to include a specific description of the processes used in ECT at the setting and provide a summary of the procedure in plain language. The peer worker could also facilitate “support groups” for individuals who are having ECT and provide support and facilitate conversation while individuals are “waiting in line” to have the procedure. Not only would this support during the waiting process help to reduce anxiety, it would also be an opportunity to provide further, or repeated, information.

#### Testing and follow up for cognitive issues

Given the significant proportion of individuals who face ongoing cognitive issues as a result of ECT, it seems prudent to provide individuals with information and options about strategies and rehabilitation opportunities if problems do arise. Information from participants in this study, participants in other studies and the experiences of the research team is that testing, follow up and rehabilitation is often difficult to source and can be very costly. It would be useful to provide access to more comprehensive cognitive testing before and after ECT and intervention options if problems emerge.

#### Greater involvement of family and friends

Greater family involvement is needed in all areas of mental health [31]. Previous studies have described initiatives to enhance family inclusion in ECT process [28]. There are numerous practical benefits to having families involved throughout the process. For many participants, families were an important source of information and support. Therefore, family members’ attitudes have a significant influence on the decision making process. Ensuring families have access to as much balanced information as possible will ensure that their attitude and advice is informed by reliable information rather than sensationalised media representations or other negative community attitudes. Additionally, because individuals receiving ECT will almost always experience difficulties retaining information from the period of time just prior to receiving ECT, family members can act as a memory aide. They could also support the individual to implement memory strategies to manage short term memory difficulties and monitor for persisting memory difficulties.

The suggestion of potentially having a family member present during the procedure is a potentially powerful practice change. Given that “fear of the unknown” is a significant driver of distress, having a family member observe the procedure may help to assuage these fears. Additionally, being able to observe the procedure would give the family member first-hand experience of the process (e.g., that it is over quickly, there is very little movement during the seizure, and there is no feared violent fitting). A clinic in the United States implemented a patient-centred strategy that involved having family members present during one or more ECT sessions [32]. A follow up focus group found that it relieved the anxiety felt by families and patients, strengthened trust in clinicians, and effectively engaged families [32]. This highlights some of the positive outcomes that may result from such an initiative. However, this is a suggestion that requires discussion and substantially more research. Family member observation of ECT is not currently undertaken anywhere in Australia and the practice is untested in the local context. It is possible that it could have unforeseen negative consequences, and perhaps even cause trauma, without careful pre-assessment of family members. It would require a major paradigm shift and substantial changes in ECT service delivery before it could be utilised in the Australian context.

### Limitations

It is important to highlight a number of potential limitations of this study. These limitations should be considered when considering the applicability of the findings from this study to other individuals and settings.

While it was not the intention of this study to determine if individuals were “for” or “against” ECT, there was a predominance of individuals who had positive experiences of ECT. This contrasts with perspectives of participants in other studies. While this study was consumer-led and all interviewers had a lived experience of ECT, many participants were advised about the study by treatment team members. Interviews were also often conducted at the hospital administering ECT. Both of these factors may have created sample bias and influenced participants to provide more positive perspectives. Additionally, many participants were at the final stages of ECT treatment or had just recently completed a course of ECT. Previous studies have suggested that individuals are more likely to report positive attitudes about ECT soon after treatment, whereas more negative opinions may emerge later [18].

Ten of the 17 participants in this study were males. As it is more common for females to receive ECT [15, 33], this could suggest that participants in this study are not representative of the demographic spread of individuals who receive ECT.

All participants were English speaking. Several potential participants were not able to be interviewed due to language barriers. This means that the opinions of individuals from culturally diverse, non-English speaking backgrounds were unable to be heard in this study. Therefore, it is not clear whether the experiences of those individuals are similar to the experiences of participants in this study.

Finally, we did not ask about whether consumers had ECT voluntarily (or not) and the type of ECT administered. If these factors influenced participants’ experiences of ECT, this would not be identified in the findings from this study.

## Conclusion

This study adds valuable further information to the emerging body of literature exploring consumers’ experiences of ECT. Despite the limitations identified above, the consumer-led nature of this study and the research team’s neutral stance (neither “for” nor “against” ECT) supports the dependability and trustworthiness of findings from this study.

To our knowledge, this is the first Australian consumer-led research to explore individuals’ experiences of ECT. Additionally, this study is one of the first to look beyond individuals’ experiences of ECT to seek suggestions to improve the decision making process and overall experience of ECT.

Recommendations emerging from this study will support more fully informed decision making and overall improved experiences for individuals who may undergo ECT. Key practice changes include providing more information, bringing up the option of ECT earlier, stronger engagement of families where relevant and providing strategies to monitor and manage potential memory and cognitive challenges that may emerge.

## Additional file

Additional file 1: Interview guide. (PDF 197 kb)

## Abbreviation

ECT: Electroconvulsive therapy
