# Supplementary material for: Decision making and support available to individuals considering and undertaking electroconvulsive therapy (ECT): a qualitative, consumer-led study
Source: BMC Psychiatry. 2018 Jul 24;18:236. doi: 10.1186/s12888-018-1813-9 (PMC6056939; doi:10.1186/s12888-018-1813-9)
Supplement: Supplementary file 1 — Interview guide. (PDF 197 kb) [file 12888_2018_1813_MOESM1_ESM.pdf]

"ECT – LET'S TALK ABOUT IT" PROJECT

Pseudonym			

Project Code			

Date of Interview			

Interviewer			

Interview Location			

Instructions:
- Ask all questions, unless otherwise stated.
- Prompts and probes are in italic under the questions. Use these to explore answers in more detail.

PARTICIPANT CHARACTERISTICS

1.1) Are you?
Male..1
Female...2
Transgender 3

1.2) How old are you? .... years

1.3) When did you first have ECT?

....

1.4) What was the treatment for?
(Prompt for depression, bipolar, etc.)

....

1.5) Which hospital was it at?

....

(For the next questions, explain 'course' as the entire block of ECT treatments. 'Individual treatments' are treatment in a single day.)

1.6) How many individual ECT treatments did you have in your first course?

....

1.7) Have you had another course of ECT treatments since your first course?
No 0
Yes..1

If 'Yes' How many courses?

....

1.3) When was your last course of ECT?

....

EXPERIENCE OF ECT

Now we want to ask you some questions about your experience with ECT. It's important we get as much detail as possible. So, we may ask you to clarify things. Remember if you feel uncomfortable with a question and do not wish to answer, you don't have to answer .

2.1) Please tell me about your first experience with ECT?

Prior

Previous other treatments
Knowledge of ECT
Discussion (health workers, family, friends, consumers, etc)
Your response to suggestion of ECT
Response of family and friends
Information provided/available (verbal discussion, written info, websites, videos)
Consent (voluntary, involuntary, who was involved)

During ECT

Expectation
Interaction with other patients
Experience with ECT sessions
Effects of treatments (good, bad, memory problems, muscle pain)

After ECT completed

Follow up after discharge (other treatments, health workers, etc)
Effects of ECT (good, bad, side effects, lasting effects, etc)
Recommend additional ECT

[If multiple courses of ECT)

2.2) Please describe your additional ECT courses.

Why additional?
Time between
How often?
How did the process compare with the first time?
What was involved in deciding?
How did the effects compare to the first time?
Do you have ongoing ECT? (maintenance)

2.3 Is there anything you would change about your previous experiences with ECT?

Information available
Consent process
Support from health workers
Support from family/friends
Support from other consumers/peers
Describe what types of support

SPECIFIC SUPPORT NEEDS FOR ECT

Now I would like to ask you about some specific areas that might have been part of the decision making process to have ECT.

3.1 Can you tell me about how you or your treating team came to the decision about having ECT?

Important considerations
Voluntary/involuntary
People who influenced the decision
Influence you had on the decision
Impact on you

3.2 Can you tell me about the information and education that you considered when you made your decision about ECT?

Healthcare workers
Resources (printed, internet, videos)
Family, friends, carers
Information in community
Information in media
Availability
Barriers
Improvement

3.2 What information, education or supports that you considered were most helpful in your decision about ECT?

Types that were helpful/not helpful
Testimonials
Procedures
Rights
What made a good resource?

3.3 Could you tell me who gave you support or encouragement with the decision to go through with ECT?

Health care workers
Family
Friends
Support provided by other ECT patients
Consumer/peer workers
Consumer/peer workers for ECT
Would any of these be useful?
How could these types of support be improved?

FINAL QUESTIONS

Now we have just a couple more questions to finish up.

4.1 From what we have talked about, can you tell me your thoughts on what are the most important areas for improving delivery of ECT for other consumers?

Information
Support
Consent
Health care workers
Community

4.2 Is there anything we haven't covered that you think is important with regards to ECT?

Thank you for your time.

(If they have given consent to be contacted about the focus groups)
We will be conducting the focus groups later in the year. These will involve discussion of new educational resources and/or consumer support programs. We will contact you to see if you would like to participate.
